# Genome-Wide Identification of Expression Quantitative Trait Loci (eQTLs) in Human Heart

**DOI:** 10.1371/journal.pone.0097380

**Published:** 2014-05-20

**Authors:** Tamara T. Koopmann, Michiel E. Adriaens, Perry D. Moerland, Roos F. Marsman, Margriet L. Westerveld, Sean Lal, Taifang Zhang, Christine Q. Simmons, Istvan Baczko, Cristobal dos Remedios, Nanette H. Bishopric, Andras Varro, Alfred L. George, Elisabeth M. Lodder, Connie R. Bezzina

**Affiliations:** 1 Department of Experimental Cardiology, Heart Failure Research Centre, Academic Medical Center, Amsterdam, The Netherlands; 2 Bioinformatics Laboratory, Department of Clinical Epidemiology, Biostatistics and Bioinformatics, Academic Medical Center, Amsterdam, The Netherlands; 3 Muscle Research Unit, Department of Anatomy, Bosch Institute, The University of Sydney, Sydney, Australia; 4 Department of Medicine, University of Miami School of Medicine, Miami, Florida, United States of America; 5 Division of Genetic Medicine, Department of Medicine, Vanderbilt University, Nashville, Tennessee, United States of America; 6 Department of Pharmacology and Pharmacotherapy, Faculty of Medicine, University of Szeged, Szeged, Hungary; 7 Department of Molecular and Cellular Pharmacology, University of Miami School of Medicine, Miami, Florida, United States of America; Institute of Metabolic Science, United Kingdom

## Abstract

In recent years genome-wide association studies (GWAS) have uncovered numerous chromosomal loci associated with various electrocardiographic traits and cardiac arrhythmia predisposition. A considerable fraction of these loci lie within inter-genic regions. The underlying trait-associated variants likely reside in regulatory regions and exert their effect by modulating gene expression. Hence, the key to unraveling the molecular mechanisms underlying these cardiac traits is to interrogate variants for association with differential transcript abundance by expression quantitative trait locus (eQTL) analysis. In this study we conducted an eQTL analysis of human heart. For a total of 129 left ventricular samples that were collected from non-diseased human donor hearts, genome-wide transcript abundance and genotyping was determined using microarrays. Each of the 18,402 transcripts and 897,683 SNP genotypes that remained after pre-processing and stringent quality control were tested for eQTL effects. We identified 771 eQTLs, regulating 429 unique transcripts. Overlaying these eQTLs with cardiac GWAS loci identified novel candidates for studies aimed at elucidating the functional and transcriptional impact of these loci. Thus, this work provides for the first time a comprehensive eQTL map of human heart: a powerful and unique resource that enables systems genetics approaches for the study of cardiac traits.

## Introduction

It is well established that many cardiac traits and susceptibility to heart disease are heritable [1], [2], [3], [4], [5], [6], [7]. Several genome-wide association studies (GWAS) have uncovered common genetic variation, in the form of single nucleotide polymorphisms (SNPs), impacting on cardiac traits such as susceptibility to atrial fibrillation [8], ventricular fibrillation [9], heart rate [10] and electrocardiographic (ECG) indices of cardiac conduction [11], [12], [13], [14] and repolarization [15], [16]. There is widespread consensus that functional studies of GWAS-defined loci will advance our understanding of the molecular underpinnings of the associated traits.

SNPs identified by GWAS are considered to impact the respective clinical phenotype, either directly or indirectly by virtue of linkage disequilibrium (LD) with the causal variant(s) in the context of a haplotype. Many trait-associated haplotypes occur in non-coding regions of the genome [17] and are hypothesized to modulate the respective trait through effects on gene expression [18]. Such SNPs are particularly challenging to understand because they may exert effects on the trait either by affecting the expression of a neighbouring gene (*cis*-effect) or the expression of a gene located elsewhere in the genome (*trans*-effects). One way of understanding GWAS signals thus entails interrogating trait-associated variants for association with differential transcript abundance by expression quantitative trait locus (eQTL) analysis. Studying gene expression level effects of disease-associated haplotypes has successfully uncovered the molecular mechanisms underlying loci associated with increased risk of myocardial infarction [19], coronary artery disease [20] and colorectal cancer [21]. In recent years, multiple genome-wide eQTL resources have become available for various tissues including brain, liver and adipose tissue [22], [23], [24], [25], [26], [27], [28], [29]. Because eQTLs may be tissue-specific, a similar resource for human heart is anticipated to have great value [23], [29], [30], [31].

To this end, we have generated a human heart eQTL resource by genome-wide genotyping and determination of transcript abundance in 129 human donor heart samples. We subsequently overlaid previously identified cardiac trait GWAS signals with the identified eQTLs to identify candidate causal genes for the effects at these GWAS loci. This work provides an eQTL map of human heart, a resource that is likely to play an important role in furthering our understanding of the mechanisms associated with loci identified in GWAS on cardiac traits.

## Results

### General design of study

We collected left ventricular samples from 180 non-diseased human hearts of unrelated organ donors whose hearts were explanted to obtain pulmonary and aortic valves for transplant surgery or explanted for heart transplantation but not used due to logistical reasons (e.g. no tissue-matched recipient was available). The subjects were assumed to be mainly of Western European descent. mRNA and DNA were isolated according to standard procedures. Transcript abundance was measured using the HumanHT-12 v4.0 whole genome array (Illumina) and genotyping was carried out using the HumanOmniExpress genome-wide SNP arrays (Illumina).

### Data preprocessing and normalization

Gene transcript abundance: Of the 47,231 transcripts whose expression levels were measured on the array, only those that were expressed above background level and for which the probe sequence mapped unambiguously to the genome and did not contain common SNPs, were used in further analyses. This procedure left 18,402 transcripts for eQTL analysis. Model-based background correction and normalization across arrays and transcripts was performed to correct for technical variance present in gene expression levels. A total of 162 arrays passed the standardized microarray gene expression quality control.

Genotyping: Manhattan distance clustering and principal component analysis of the genotype data of 154 samples that were successfully genotyped, revealed 13 genetic outliers (**Figure S1**). To ensure a genetically homogenous group for further analysis, samples pertaining to these clusters were removed. An additional 12 samples were removed due to low call rate (<95%), high proportion of alleles identical-by-state (>95%), or extreme heterozygosity (FDR 1%). Only SNPs with a minor allele frequency (MAF) higher than 0.15 were considered in eQTL analysis. This cutoff was chosen to ensure sufficient power to detect eQTLs within a broad range of effect sizes (**Figure S2**). Imputation was performed using the HAPMAP Phase III data (see Materials & Methods for details). This left 129 samples (74 male, 55 female; age 41±14), 18,402 transcripts and 897,683 SNPs for eQTL analysis.

### Genome-wide eQTL mapping

Each of the measured transcripts was tested for association with all SNPs using linear modeling, taking age, sex and clinical/university center as covariates. We thus identified 6402 significant eQTLs (FDR ≤0.05). To remove redundant signals and identify independent expression-controlling loci, we performed linkage-disequilibrium (LD)-pruning. For this we grouped SNPs exhibiting LD (r^2^>0.6) into clusters, revealing 771 independent loci regulating 429 unique transcripts. These results are comparable to eQTL studies in other non-diseased tissues of similar sample size [22], [23], [24], [28], [29].

**Figure 1:**
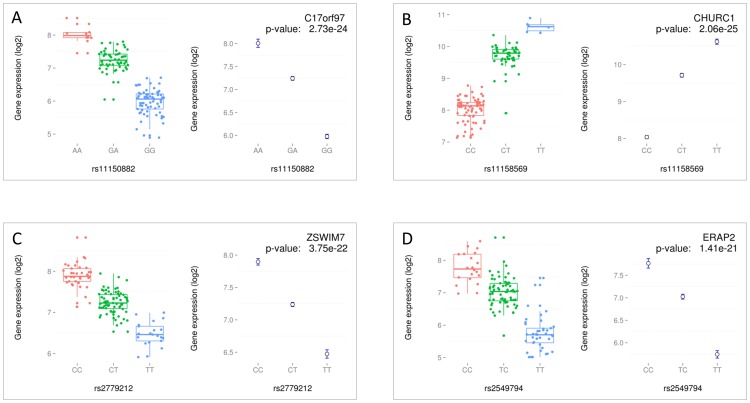
Overview plots for top *cis* eQTLs. An overview of the 4 most significant *cis* eQTLs: rs11150882 with *C17orf97* (panel A), rs11158569 with *CHURC1* (panel B), rs2779212 with *ZSWIM7* (panel C) and rs2549794 with *ERAP2* (panel D). On the left of each panel, box-and-whisker plots of mRNA levels for all genotypes. On the right, mean and standard-error plots of mRNA levels for all genotypes are illustrated. Right upper corner gives the association p-value and the gene name.

Of these 771 eQTLs, 770 were *cis*-eQTLs for 428 unique transcripts (p<2.82×10^−5^; FDR ≤0.05), where the associated SNPs lie within 1 Mb of the transcriptional start site (TSS) of the cognate transcript. For the four most significant *cis*-eQTLs, box-and-whisker plots and mean-standard-error plots for the individual genotypes are given in **Figure 1**. An overview of the most significant *cis*-eQTLs is given in **Table 1** and the complete results are given in supplemental **Table S1**.

**Table 1:** Overview of the 30 most significant *cis* eQTLs, reported as independent LD-pruned SNP clusters (see Materials & Methods).

LD cluster	Top SNP ID	Chr.	SNP position	Gene TSS position	Gene strand	Gene symbol	eQTL p-value	Minor allele	Major allele	eQTL beta	eQTL MAF	Distance top SNP to gene	Relative top SNP position	Illumina Probe ID
1	*rs11158569*	14	65400069	65381079	+	*CHURC1*	2.06E-25	T	C	1.50	0.24	0	*inside*	*ILMN_1798177*
2	*rs11150882*	17	259648	260118	+	*C17orf97*	2.73E-24	A	G	1.11	0.29	−470	*upstream*	*ILMN_1707137*
3	*rs2779212*	17	15876655	15903006	−	*ZSWIM7*	3.75E-22	T	C	−0.70	0.44	3220	*downstream*	*ILMN_3298167*
4	*rs2549794*	5	96244549	96211644	+	*ERAP2*	1.41E-21	C	T	1.06	0.42	0	*inside*	*ILMN_1743145*
5	*rs335632*	5	76728085	76788332	−	*WDR41*	4.44E-20	G	A	−0.87	0.17	0	*inside*	*ILMN_1778488*
6	*rs1051470*	12	118583232	118573870	+	*PEBP1*	1.74E-19	T	C	−1.70	0.38	0	*inside*	*ILMN_3285785*
7	*rs4837796*	9	123610288	123605320	+	*LOC253039*	2.36E-19	G	A	0.69	0.38	0	*inside*	*ILMN_3236498*
8	*rs12358834*	10	29778270	30024730	−	*SVIL*	2.83E-19	C	A	−1.37	0.16	0	*inside*	*ILMN_3298400*
9	*rs8413*	9	139323311	139334256	−	*INPP5E*	1.45E-18	G	A	−0.60	0.40	0	*inside*	*ILMN_1811301*
10	*rs7909832*	10	16556710	16478942	+	*PTER*	2.78E-18	A	G	−0.74	0.45	966	*downstream*	*ILMN_1795336*
11	*rs11586488*	1	22348556	22351707	+	*HSPC157/LINC00339*	8.56E-18	C	T	1.01	0.23	−3151	*upstream*	*ILMN_3272768*
12	*rs4822466*	22	24312204	24384284	−	*GSTT1*	1.10E-17	G	A	−2.04	0.45	63935	*downstream*	*ILMN_1730054*
12	*rs5742303*	22	24299147	24309026	+	*DDTL*	3.57E-15	C	T	0.47	0.42	−9879	*upstream*	*ILMN_3244439*
12	*rs5742303*	22	24299147	24236565	+	*MIF*	9.86E-06	C	T	0.17	0.42	61738	*downstream*	*ILMN_1807074*
13	*rs7168431*	15	41672384	41694658	−	*NDUFAF1*	1.43E-17	G	A	−0.63	0.28	7163	*downstream*	*ILMN_1754421*
14	*rs1603117*	4	70395945	69962193	+	*UGT2B7*	7.49E-17	G	A	−1.41	0.37	417240	*downstream*	*ILMN_1679194*
14	*rs1603117*	4	70395945	69434245	−	*UGT2B17*	2.25E-16	G	A	−1.21	0.37	−961700	*upstream*	*ILMN_1808677*
14	*rs1603117*	4	70395945	70080449	−	*UGT2B11*	3.64E-14	G	A	−0.98	0.37	−315496	*upstream*	*ILMN_1810233*
14	*rs1603117*	4	70395945	70361626	−	*UGT2B4*	5.77E-11	G	A	−0.67	0.37	−34319	*upstream*	*ILMN_2206500*
15	*rs10876864*	12	56401085	56435686	+	*RPS26/L/P10*	8.22E-16	G	A	0.46	0.36	−34601	*upstream*	*ILMN_3290019*
16	*rs720201*	2	61376463	61372243	+	*C2orf74*	1.06E-15	G	A	0.61	0.42	0	*inside*	*ILMN_1754501*
17	*rs4796398*	17	7208197	7210318	+	*EIF5A*	1.39E-15	G	A	0.34	0.39	−2121	*upstream*	*ILMN_1794522*
18	*rs1222809*	5	79917517	79950800	−	*DHFR*	2.00E-15	G	A	−0.92	0.22	4528	*downstream*	*ILMN_3232696*
19	*rs530411*	19	39927540	39926618	−	*RPS16*	4.43E-15	A	G	0.20	0.37	−922	*upstream*	*ILMN_1651850*
20	*rs10051931*	5	94001476	93954391	+	*ANKRD32*	8.79E-15	G	A	−0.47	0.33	0	*inside*	*ILMN_2214278*
21	*rs1887547*	1	110295772	110283660	−	*GSTM3*	1.21E-14	A	G	−0.65	0.36	−12112	*upstream*	*ILMN_1736184*
22	*rs2240147*	19	989730	984328	+	*WDR18*	1.95E-14	A	C	0.42	0.18	0	*inside*	*ILMN_1694479*
23	*rs10088428*	8	28909523	28747911	+	*HMBOX1*	2.14E-14	A	G	−0.52	0.24	0	*inside*	*ILMN_1720059*
24	*rs113413*	22	24292264	24384284	−	*GSTT1*	3.05E-14	G	A	−2.25	0.39	83875	*downstream*	*ILMN_1730054*
24	*rs113413*	22	24292264	24309026	+	*DDTL*	5.74E-12	G	A	0.49	0.39	−16762	*upstream*	*ILMN_3244439*
25	*rs9568*	15	41573612	41694658	−	*NDUFAF1*	3.71E-14	A	C	−0.59	0.27	105935	*downstream*	*ILMN_1754421*
26	*rs4431401*	6	86189520	86388451	−	*C6orf160*	4.41E-14	T	C	−0.54	0.48	197205	*downstream*	*ILMN_1653794*
27	*rs11007559*	10	29698286	30024730	−	*SVIL*	6.04E-14	A	C	−1.02	0.21	47991	*downstream*	*ILMN_3298400*
28	*rs6663*	12	109886603	109915155	−	*KCTD10*	1.00E-13	A	G	0.55	0.26	0	*inside*	*ILMN_1719064*
29	*rs2395943*	6	42940673	42946981	−	*PEX6*	1.05E-13	A	G	−0.66	0.43	0	*inside*	*ILMN_1683279*
30	*rs11800014*	1	22414070	22351707	+	*HSPC157/LINC00339*	2.04E-13	A	G	0.97	0.16	56355	*downstream*	*ILMN_3272768*

The MAF and beta (effect size per copy of the minor allele) for the most significant SNP of each cluster is listed. LD  =  linkage disequilibrium, TSS  =  transcription start site, Chr.  =  chromosome.

Of the independent significant eQTLs, one was found to be in *trans* (p<2.12×10^−11^; FDR ≤0.05), with the expression of *LOC644936* located on chromosome 5 being seemingly modulated by an eQTL (rs852423) on chromosome 7. However, as *LOC644936* is a known pseudogene of *ACTB* and rs852423 is located within *ACTB*, we cannot rule out the possibility that rs852423 is in fact a *cis* eQTL for *ACTB* rather than a *trans* eQTL for LOC644936. Using BLAST to align the microarray probe sequence of *LOC644936* to the human transcriptome uncovered a partial match with *ACTB* in addition to a 100% match with *LOC644936*.

### Integration of eQTL data with cardiac GWAS loci

**Figure 2:**
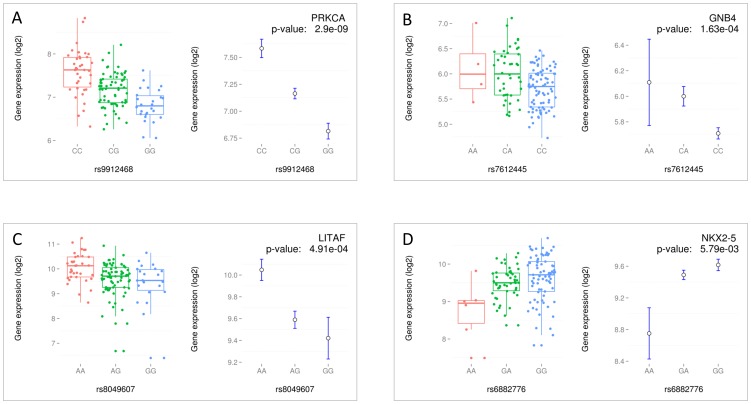
eQTL overview plots for 4 cardiac trait GWAS candidate genes. An overview of 4 GWAS *cis* eQTLs: rs9912468 with *PRKCA* (panel A), rs7912445 with *GNB4* (panel B), rs8049607 with *LITAF* (panel C) and rs6882776 with *NKX2-5* (panel D). On the left of each panel, box-and-whisker plots of mRNA levels for all genotypes. On the right, mean and standard-error plots of mRNA levels for all genotypes are illustrated. Right upper corner gives the association p-value and the gene name.

In order to provide candidate genes for the reported heart-related GWAS loci, we listed the 102 SNPs previously associated with a cardiac trait at genome-wide statistical significance (p_gwas_ ≤5×10^−8^), representing 74 independent loci (LD-pruned with r^2^>0.6, see Materials & Methods). These corresponded to loci associated with ventricular fibrillation/sudden cardiac death, atrial fibrillation, heart rate, PR interval, QRS duration and QTc interval. Of these, the 64 SNPs that displayed a MAF of 15% or higher in the eQTL sample were overlaid with the eQTL data to identify transcripts under genetic regulation by these loci. All GWAS SNPs were tested for association with transcript levels of all 18,402 transcripts in this study. We identified a *cis* association between rs9912468, a modulator of QRS duration [12] with the level of expression of the *PRKCA* transcript at genome-wide statistical significance (p = 2.90×10^−9^, see **Figure 2A**). Besides *PRKCA*, no other GWAS SNP displayed an eQTL association p-value that passed the stringent Bonferroni-corrected p-value threshold (p<0.05/64 SNPs ×18,402 transcripts ∼ 4×10^−8^). A total of 34 SNPs were associated with the transcript level of a gene at a p≤0.05 (**Table 2**). Among these, rs8049607, a modulator of QTc-interval [16] was found to be associated in *cis* with the transcript level of *LITAF* (p<5×10^−4^, **Figure 2C**), and rs7612445 and rs6882776, both associated with heart rate [10] were associated in *cis* with the transcript levels of *GNB4* (p<2×10^−4^, **Figure 2B**) and *NKX2-5* (p<6×10^−3^, **Figure 2D**), respectively. The number of nominal associations for the 64 cardiac trait-associated SNPs tested represents a more than 7-fold enrichment (p<0.05, see Materials & Methods) compared to a random selection of 64 variants from the entire set of SNPs used in eQTL analysis.

**Table 2:** Look-up of SNPs from cardiac GWAS in eQTL data.

LD cluster	SNP	Chr.	SNP Position	eQTL gene TSS position	eQTL Gene Symbol	eQTL p-value	eQTL beta	eQTL MAF	GWAS trait	Reported GWAS candidate gene	Candidate gene was measured	References
1	*rs9912468*	17	64318357	64298926	*PRKCA*	2.90E-09	−0.37	0.45	QRS duration	*PRKCA*	Y	[12]
1	*rs9912468*	17	64318357	65241319	*HELZ*	1.08E-02	−0.16	0.45	QRS duration	*PRKCA*	Y	*idem*
2	*rs7612445*	3	179172979	179169371	*GNB4*	1.63E-04	0.28	0.20	Heart rate	*GNB4*	Y	[10]
2	*rs7612445*	3	179172979	179280708	*ACTL6A*	2.69E-02	0.11	0.20	Heart rate	*GNB4*	Y	*idem*
2	*rs7612445*	3	179172979	178866311	*PIK3CA*	3.77E-02	−0.11	0.20	Heart rate	*GNB4*	Y	*idem*
3	*rs8049607*	16	11691753	11681322	*LITAF*	4.91E-04	−0.32	0.45	QTc duration	*LITAF,CLEC16A, SNN, ZC3H7A, TNFRSF17*	Y, N, Y, Y, N	[15], [16]
4	*rs10824026*	10	75421208	75255782	*PPP3CB*	3.00E-02	−0.10	0.17	Atrial fibrillation	*SYNPO2L*	Y	[56]
4	*rs10824026*	10	75421208	74870210	*NUDT13*	3.94E-02	−0.13	0.17	Atrial fibrillation	*SYNPO2L*	Y	*idem*
5	*rs2968864*	7	150622162	151574316	*PRKAG2*	1.0E-03	0.13	0.22	QTc duration	*KCNH2*	N	*idem*
5	*rs2968864*	7	150622162	150020758	*ACTR3C*	1.49E-03	−0.11	0.23	QTc duration	*KCNH2*	N	[15], [16]
5	*rs2968864*	7	150622162	150026938	*C7orf29*	3.69E-02	0.09	0.23	QTc duration	*KCNH2*	N	*idem*
6	*rs223116*	14	23977010	24912007	*SDR39U1*	3.57E-03	0.16	0.26	Heart rate	*MYH7, NDNG*	N, N	[57]
6	*rs223116*	14	23977010	24424298	*C14orf167*	4.06E-03	0.19	0.26	Heart rate	*MYH7, NDNG*	N, N	*idem*
6	*rs223116*	14	23977010	23938898	*NGDN*	1.45E-02	−0.10	0.26	Heart rate	*MYH7, NDNG*	N, N	*idem*
6	*rs223116*	14	23977010	24912007	*C14orf124*	1.69E-02	0.23	0.26	Heart rate	*MYH7, NDNG*	N, N	*idem*
6	*rs223116*	14	23977010	24605378	*PSME1*	3.23E-02	0.09	0.26	Heart rate	*MYH7, NDNG*	N, N	*idem*
6	*rs223116*	14	23977010	24701648	*GMPR2*	3.37E-02	0.09	0.26	Heart rate	*MYH7, NDNG*	N, N	*idem*
7	*rs7784776*	7	46620145	45927959	*IGFBP1*	5.71E-03	0.08	0.43	QRS duration	*IGFBP3*	Y	[12]
8	*rs6882776*	5	172664163	172662315	*NKX2-5*	5.79E-03	−0.24	0.24	Heart rate	*NKX2-5*	Y	[10]
8	*rs6882776*	5	172664163	172261223	*ERGIC1*	2.23E-02	−0.14	0.24	Heart rate	*NKX2-5*	Y	*idem*
9	*rs12143842*	1	162033890	161169105	*NDUFS2*	6.44E-03	0.15	0.28	QTc duration	*OLFML2B, NOS1AP*	N, Y	[15], [16], [58]
9	*rs12143842*	1	162033890	161147758	*B4GALT3*	2.17E-02	−0.10	0.28	QTc duration	*OLFML2B, NOS1AP*	N, Y	*idem*
10	*rs6800541*	3	38774832	39149130	*GORASP1*	7.17E-03	0.12	0.41	PR duration	*SCN10A*	N	[11]
10	*rs6801957*	3	38767315	39149130	*GORASP1*	8.17E-03	0.12	0.41	PR duration	*-*	-	[59]
10	*rs6801957*	3	38767315	39149130	*GORASP1*	8.17E-03	0.12	0.41	QRS duration	*SCN10A*	N	[12]
10	*rs6795970*	3	38766675	39149130	*GORASP1*	9.64E-03	0.12	0.40	PR duration	*SCN10A*	N	[13], [14]
10	*rs6795970*	3	38766675	39149130	*GORASP1*	9.64E-03	0.12	0.40	QRS duration	*SCN10A*	N	[14]
10	*rs6599250*	3	38784029	39149130	*GORASP1*	1.23E-02	0.12	0.41	PR duration	*-*	-	[59]
10	*rs6599254*	3	38795555	39149130	*GORASP1*	1.23E-02	0.12	0.41	PR duration	*-*	-	*idem*
11	*rs13030174*	2	232271284	231989824	*HTR2B*	7.78E-03	−0.15	0.22	Heart rate	*B3GNT7*	Y	[10]
12	*rs13165478*	5	153869040	153825517	*SAP30L*	8.72E-03	0.13	0.36	QRS duration	*HAND1-SAP30L*	N	[12]
12	*rs13165478*	5	153869040	154317776	*GEMIN5*	1.47E-02	0.12	0.36	QRS duration	*HAND1-SAP30L*	N	*idem*
13	*rs2242285*	3	66431602	66119285	*SLC25A26*	8.94E-03	0.19	0.47	QRS duration	*LRIG1-SLC25A26*	N	*idem*
14	*rs7562790*	2	36673555	36582713	*LOC100288911*	1.04E-02	−0.20	0.40	QRS duration	*CRIM1*	N	*idem*
15	*rs2074518*	17	33324382	33307517	*LIG3*	1.1E-03	0.15	0.48	QTc duration	*LIG3,RFFL*	Y,N	[15]
15	*rs2074518*	17	33324382	34136459	*TAF15*	3.6E-02	0.14	0.48	QTc duration	*LIG3,RFFL*	Y,N	*idem*
15	*rs2074518*	17	33324382	33885110	*SLFN14*	4.2E-02	−0.04	0.48	QTc duration	*LIG3,RFFL*	Y, N	*idem*
16	*rs13376333*	1	154814353	153963239	*RPS27*	2.66E-02	−0.20	0.31	Atrial fibrillation	*KCNN3*	Y	[8]
16	*rs13376333*	1	154814353	153958806	*RAB13*	4.22E-02	0.09	0.31	Atrial fibrillation	*KCNN3*	Y	*idem*
17	*rs7433723*	3	38784957	39149130	*GORASP1*	1.18E-02	0.12	0.42	PR duration	*-*	-	[59]
18	*rs3922844*	3	38624253	38537763	*EXOG*	1.34E-02	0.15	0.32	PR duration	*SCN5A*	Y	*idem*
19	*rs365990*	14	23861811	23398661	*PRMT5*	1.49E-02	−0.09	0.40	Heart rate	*MYH6*	Y	[10], [14]
19	*rs452036*	14	23865885	23398661	*PRMT5*	1.49E-02	−0.09	0.40	Heart rate	*MYH6*	Y	[57]
19	*rs365990*	14	23861811	24711880	*TINF2*	1.81E-02	−0.13	0.40	Heart rate	*MYH6*	Y	[10], [14]
19	*rs365990*	14	23861811	23340960	*LRP10*	2.22E-02	−0.07	0.40	Heart rate	*MYH6*	Y	*idem*
19	*rs452036*	14	23865885	23340960	*LRP10*	2.22E-02	−0.07	0.40	Heart rate	*MYH6*	Y	[57]
19	*rs365990*	14	23861811	23526747	*CDH24*	2.70E-02	−0.09	0.40	Heart rate	*MYH6*	Y	[10], [14]
19	*rs452036*	14	23865885	23526747	*CDH24*	2.70E-02	−0.09	0.40	Heart rate	*MYH6*	Y	[57]
20	*rs2824292*	21	18787176	18985268	*BTG3*	1.96E-02	−0.19	0.48	Sudden cardiac death	*CXADR, BTG3*	N, Y	[9]
21	*rs13245899*	7	100497131	100797686	*AP1S1*	2.01E-02	−0.15	0.18	Heart rate	*ACHE*	Y	[10]
21	*rs314370*	7	100453208	99933688	*PILRB*	2.40E-02	−0.08	0.17	Heart rate	*SLC12A9*	Y	[57]
21	*rs13245899*	7	100497131	99933688	*PILRB*	2.47E-02	−0.08	0.18	Heart rate	*ACHE*	Y	[10]
21	*rs13245899*	7	100497131	99717481	*TAF6*	4.12E-02	−0.13	0.18	Heart rate	*ACHE*	Y	*idem*
21	*rs314370*	7	100453208	100797686	*AP1S1*	4.74E-02	−0.13	0.17	Heart rate	*SLC12A9*	Y	[57]
22	*rs1321311*	6	36622900	36164550	*BRPF3*	2.02E-02	0.10	0.28	QRS duration	*CDKN1A*	N	[14]
23	*rs885389*	12	131621762	131323819	*STX2*	1.1E-02	0.09	0.35	Heart rate	*GPR133*	N	[10]
24	*rs4657178*	1	162210610	161520413	*FCGR3A*	2.44E-02	−0.10	0.23	QTc duration	*NOS1AP*	Y	[60]
25	*rs1152591*	14	64680848	65569227	*MAX*	2.50E-02	0.14	0.49	Atrial fibrillation	*SYNE2*	N	[56]
26	*rs7980799*	12	33576990	34175216	*ALG10*	2.63E-02	−0.06	0.43	Heart rate	*SYT10*	Y	[10]
27	*rs4725982*	7	150637863	150020296	*LRRC61*	2.78E-02	−0.07	0.24	QTc duration	*KCNH2*	N	[15], [16]
27	*rs4725982*	7	150637863	151038847	*NUB1*	4.88E-02	0.06	0.24	QTc duration	*KCNH2*	N	*idem*
28	*rs727957*	21	35880072	34915198	*GART*	3.39E-02	0.13	0.17	QTc duration	*KCNE1*	Y	[14]
29	*rs12498374*	4	111584419	110481355	*CCDC109B*	4.54E-02	−0.21	0.23	Atrial fibrillation	*-*	-	[61]
30	*rs7312625*	12	114799974	114846000	*LOC255480*	4.88E-02	−0.05	0.26	PR duration	*TBX5*	Y	[59]
31	*rs826838*	12	39106731	39299420	*CPNE8*	1.3E-02	0.14	0.42	Heart rate	*CPNE8*	Y	[10]
31	*rs826838*	12	39106731	39837192	*KIF21A*	3.8E-02	−0.09	0.422481	Heart rate	*CPNE8*	Y	*idem*
32	*rs6127471*	20	36844038	37434348	*PPP1R16B*	3.3E-02	0.16	0.46	Heart rate	*KIAA1755*	N	*idem*
33	*rs2067615*	12	107149422	107168399	*RIC8B*	4.8E-02	0.09	0.47	Heart rate	*RFX4*	Y	*idem*
34	*rs4074536*	1	116310967	115632121	*TSPAN2*	1.05E-02	−0.08	0.28	QRS duration	*CASQ2*	Y	[12]

Overview of eQTL effects of reported cardiac electric trait related GWAS SNPs. Only GWAS SNPs reaching genome-wide significance as stated in the original studies (p-value ≤5×10^−8^) and with nominal eQTL association (p≤0.05) are reported. This resulted in 34 independent loci. *PRKCA* (*rs9912468*, QRS duration) reaches genome-wide significance (4×10^−8^; represented in bold in table). The beta is defined as the effect size per copy of the minor allele. LD  =  linkage disequilibrium, TSS  =  transcription start site, Chr.  =  chromosome, Y  =  yes, N  =  no.

### Discussion

We conducted a genome-wide eQTL analysis in 129 samples of normal human myocardium, identifying genetic variation regulating gene expression in human heart and uncovering 771 genome-wide significant independent eQTLs. This resource, heretofore unavailable in human heart will contribute to advancing our understanding of the genetic mechanisms underlying loci associated with cardiac traits. All but one of the eQTLs identified were *cis* eQTLs. Other eQTL studies have identified only few *trans* eQTLs [22], [24], [28], [29], illustrating the general difficulty of detecting *trans*-regulatory variants in eQTL studies [31], [32]. Based on larger eQTL studies in other tissues [22], [24], [25], [26], [29] as many as 4000 independent cardiac *cis* eQTLs are expected to be present, hence the results presented here are a subset of this theoretical complete set of cardiac eQTLs.

In recent years, many novel loci associated with a number of cardiac traits, including cardiac arrhythmia and ECG indices, have been discovered. However, the identification of (novel) genes at these loci has lagged behind. The availability of a cardiac eQTL resource is likely to aid in the dissection of these loci by providing a means of prioritizing candidate genes for follow-up functional studies. Indeed, our current findings already provide candidate genes for a number of these loci (**Table 2**). One such example is the *PRKCA* gene for the effect observed on QRS duration for the rs9912468-tagged haplotype on chromosome 9. *PRKCA* encodes protein kinase C alpha, a fundamental regulator of cardiac contractility and Ca^2+^ handling in cardiomyocytes [33]. The mechanism by which it regulates QRS duration is unknown. Other candidates include the *LITAF* gene (encoding lipopolysaccharide-induced TNF factor) for the rs8049607-tagged haplotype associated with QTc-interval and the *GNB4* gene (encoding guanine nucleotide binding protein) for the rs7612445-tagged haplotype associated with heart rate. None of these eQTLs (for *PRKCA, LITAF* and *GNB4*) have been previously identified in non-cardiac tissues.

The utility of this approach is further evidenced by the fact that the 64 GWAS SNPs were enriched in nominally significant eSNPs as compared to a random selection of 64 variants from the entire set of SNPs used in eQTL analysis. Such an enrichment was reported before for GWAS loci in general based on eQTLs identified in lymphoblastoid cell lines from HAPMAP samples [18].

The eQTLs we identified represent an enriched set of highly relevant candidates to test in future studies for association with cardiac traits and disease. Among the highly significant eQTLs listed in **Table 1**, at least two SNPs could also be interesting from a pharmacogenetic point of view. One is rs1222809 which was found to be strongly associated with the expression level of the *DHFR* gene encoding dihydrofolate reductase, a putative target of the drug methotrexate. Of note previous studies have provided evidence that rs1650697, which is in complete LD with rs1222809, may be associated with adverse events to methotrexate in patients with rheumatoid arthritis [34], [35]. The other potentially interesting eQTL from a pharmacogenetic point of view is rs4822466 which was found to be highly associated with the expression of *GSTT1*, a gene encoding the liver detoxifying enzyme Glutathione S-transferase T1.

The eQTLs we identified are expected to be enriched in the regulatory regions of the genome such as promoter regions, enhancers and transcription factor binding sites [36]. Recent work has begun to uncover these relationships for adult human heart [37]. However, formal testing for enrichment of eQTLs in the known regulatory regions [37] did not provide statistically significant enrichment (data not shown). At least in part, this may be due to the limited number of eQTLs we have identified.

A limitation of the presented study concerns the fact that not all transcripts have been tested for eQTL effects. Transcripts that were expressed below the (array-based) detection level or for which probe design was not optimal could not be tested. Conversely, not all haplotypes in the genome were tested as for instance we only tested SNPs with a MAF higher than 0.15. Furthermore, our sample size and therefore statistical power was limited, preventing the identification of eQTLs of smaller effect and *trans* eQTLs. The interpretation of the data concerning SNPs from GWAS presented in **Table 2** must take these considerations into account. Additionally, the single *trans* eQTL we identified is likely a false discovery and will require further investigation.

Our study was conducted in left ventricular myocardium. However, it is well known that different cardiac compartments such as the atria or the specialized conduction system display different gene expression patterns [38], [39], [40], [41] and eQTL effects might thus differ across cardiac compartments. Furthermore, we have no information relating to cardiac traits such as ECG indices in the 129 individuals from whom the left ventricular samples were obtained; we were therefore unable to correlate gene expression with cardiac traits in these individuals [23], [42].

In summary, we here provide the first eQTL map of human left ventricular myocardium that will enable systems genetics approaches in the study of cardiac traits.

## Materials and Methods

### Ethics statement

Investigations using the human ventricular samples conformed to the principles outlined in the Helsinki Declaration of the World Medical Association. The ethical review boards of University of Szeged (Ethical Review Board of the University of Szeged Medical Center; Szeged, Hungary), Vanderbilt University (Institutional Review Board of Vanderbilt University School of Medicine; Nashville, USA), University of Miami (Institutional Review Board of the University of Miami School of Medicine; Miami, USA), and the University of Sydney (Human Research Ethics Committee (HREC); Sydney, Australia) approved procurement and handling of the human cardiac material. Written informed consent from the donor or the next of kin was obtained for use of this sample in research. All data was analyzed anonymously.

### Sample collection

Left ventricular samples were obtained from 180 non-diseased human hearts of unrelated organ donors whose hearts were explanted to obtain pulmonary and aortic valves for transplant or valve replacement surgery or explanted for transplantation but not used due to logistical reasons. The tissues were ascertained at the University of Szeged (Hungary; n = 79), Vanderbilt University (Nashville, USA; n = 46), University of Miami (USA; n = 30), and the University of Sydney (Australia; n = 25) and assumed to consist mainly of subjects of Western European descent based on self-reported ethnicity. The Vanderbilt samples were procured with the assistance of the National Disease Research Interchange (Philadelphia, PA).

### Generation and processing of gene expression data

Total RNA was extracted from the human left ventricular heart samples using the *mir*Vana miRNA isolation kit (Ambion) at the AMC, Amsterdam, The Netherlands. Sample processing order was randomized. RNA quality was assessed by Agilent Bioanalyzer (minimum RIN = 7) and spectrophotometry (minimum 260 nm:280 nm = 1.8). The Illumina TotalPrep-96 RNA Amplification Kit was used to generate cRNA starting from 200 ng total RNA. Genome-wide gene expression data was generated using Illumina HumanHT-12 v4 BeadArrays, containing 47,231 probes representing 28,688 RefSeq annotated transcripts (ServiceXS, Leiden, The Netherlands), following the instructions of the manufacturer.

Raw expression data were imported into the Illumina BeadStudio and summarized at probe-level for each sample without normalization or background correction. The summarized data were subsequently imported into R (version 2.15.3) [43] using the *beadarray* package [44]. Quality control was performed using the ArrayQualityMetrics package in R [45]. Samples displaying transcriptional stratification using hierarchical clustering were omitted from the analysis. The summarized data of the 162 remaining samples was background corrected and quantile normalized using the *neqc* algorithm [46] across all samples. The *neqc* algorithm is the current standard data-preprocessing method for Illumina gene expression BeadArrays [47], and has been applied in eQTL studies with comparable sample size [29], [30].

Probes containing common SNPs (HAPMAP Phase III release 2) [27], [29] and probes whose sequence did not align or aligned ambiguously to the human reference genome (HG19), according to up-to-date Illumina HumanHT-12 v4.0 BeadArray annotation available from the Bioconductor project, were left out of the analysis. Additionally, probes with median expression levels below a study specific threshold (the median expression levels of Y chromosome transcripts in the female subjects of the sample population) were not considered for subsequent analyses.

### Genotyping and genotype imputation

DNA was extracted for genotyping from 162 heart samples that passed the gene expression analysis quality control criteria (see above) at the AMC, Amsterdam, The Netherlands. Genome-wide SNP genotyping was carried out using Illumina HumanOmniExpress Beadchips interrogating 733,202 genetic markers (Genome Analysis Center, Helmholtz Zentrum München, Germany). A total of 8 samples had sample quality issues (and were not hybridized) or failed hybridization, leaving genotype data for 154 samples. Quality control was performed in the *GenABEL* [48] package in R using default settings. Samples with low call rate (<95%), extreme heterozygosity (FDR 1%) or high proportion of alleles identical-by-state (>95%) were removed. Additionally, any remaining samples showing genetic stratification through Manhattan distance hierarchical clustering (using the *popgen* [49] package in R), and confirmed with principal component analysis [48], were not considered (**Figure S1**).

Power calculations were performed (with a fixed FDR of 0.05) to assess the influence of MAF on power in relation to observed gene expression fold changes. Based on these results, a MAF threshold of 0.15 was chosen to ensure sufficient power to detect *cis* eQTLs within a broad range of effect sizes (**Figure S2**). Additionally, assuming Hardy-Weinberg equilibrium, a MAF of 0.15 or higher yields an expected number of three individuals homozygous for the minor allele, which we considered the minimum for fitting a meaningful additive genetic model.

Imputation was performed using the MACH software [50] and the HAPMAP Phase III data. Only SNPs imputed with sufficient confidence were considered, using the estimate of the squared correlation between imputed and true genotypes. By setting the cut-off at 0.30, most of the poorly imputed SNPs are filtered out, compared to only a small number (<1%) of well imputed SNPs [51].

### eQTL statistical analysis

After pre-processing and stringent quality control of gene expression and genotypic data as described above, a total of 129 heart samples were used in eQTL analysis. Each transcript was tested for association with SNP genotypes genome-wide using linear modeling (assuming an additive genetic model), taking age, gender and tissue collection center as covariates, using the *GenABEL* package [48] in R. Correction for multiple testing was performed on the complete set of *cis* eQTL p-values in the *qvalue* package in R [52]. A q-value (FDR) ≤0.05 was considered significant for *cis* eQTLs, corresponding to a p-value of 2.82×10^−5^. *Cis* relations were defined as those within 1 Mb of a transcription start site (TSS), in accordance with previous reports demonstrating that over 90% of *cis* SNPs are situated within 100 Kb of a TSS [26], [27], [29], [47], [53]. SNPs with an LD *R^2^* of larger than 0.6 were considered dependent and LD-pruned into clusters (LD clusters), in accordance with previous studies [23], [29], [30]. For *trans* eQTLs, only results with a p-value <5×10^−8^ were considered (corresponding to a target α (or p value) of 0.05 with a Bonferroni correction for 1 million independent tests [54], [55]). Correction for multiple testing was done by using a step-up Benjamini & Hochberg procedure on all p-values <5×10^−8^, and a q-value (FDR) ≤0.05 was considered genome-wide significant for *trans* eQTLs, corresponding to a p-value of 2.12×10^−11^.

### eQTL biological interpretation and candidate gene prioritization

To prioritize candidate genes for further studies, additional data sources were integrated. Additional trait and disease associated SNPs were extracted from PubMed (www.ncbi.nlm.nih.gov/pubmed; search terms: ‘GWAS’ AND ‘cardiac’, ‘atrial fibrillation’, ‘sudden cardiac death’, ‘ECG [electrocardiographic]’, ‘PR interval’, ‘QRS’, ‘QT’, ‘repolarization’), the NHGRI catalog of published GWAS (http://www.genome.gov/gwastudies/), and GWAS central (https://www.gwascentral.org) on January 8, 2013. Analyses were restricted to samples of European ancestry. Results were classified into six categories: sudden cardiac death, atrial fibrillation, heart rate, PR duration, QRS duration and QTc duration. Next, each GWAS SNP passing genome-wide significance in the respective study (5×10^−8^, a target α of 0.05 with a Bonferroni correction for 1 million independent tests) was tested for association with expression of all 18,402 measured transcripts. To determine the number of independent loci, LD-pruning was performed by merging all GWAS SNPs with LD r^2^>0.6 (HAPMAP R22 and HAPMAP Phase III). The p-value threshold for significant eQTL effects was set at 4×10^−8^, a target α of 0.05 with a Bonferroni correction for 1,177,728 tests (64 independent loci ×18,402 transcripts).

To quantify the enrichment of eQTLs among the cardiac trait GWAS SNPs, we generated 100,000 randomized independent SNP sets of the same size as the number of independent GWAS loci, and with corresponding MAF distribution and proximity to genes. The number of nominally significant eQTL associations for the original independent GWAS loci is referred to as Q. Next, for each random set S_i_, we determined the number of eQTLs at nominal significance (p≤0.05), referred to as Q_i_. The simulations yielded a fold-enrichment score, calculated as the average over all random sets of the ratio between Q and Q_i_, and an empirical p-value, calculated as the proportion of simulations in which the number of eQTLs exceeds the number of nominally significant eQTL associations in the original independent GWAS loci.

### Public access to microarray data

The microarray genotyping and gene expression data of the study have been deposited online at the Gene Expression Omnibus (GEO), with accession number GSE55232.

## Supporting Information

Figure S1 **Manhattan distance hierarchical clustering dendogram of 154 genotyped subjects.** Manhattan distance hierarchical clustering revealed several genotypic outliers. The clustering was repeated using principal component analysis, identifying the same groups of outliers.
(TIF)

Figure S2 **Results of eQTL power analyses in relation to MAF and gene expression fold change.** eQTL power analyses were performed for different minimum minor allele frequencies (0.05, 0.10, 0.15, 0.20, 0.30 and 0.40). The gene expression fold change is defined as log_2_ difference in gene expression observed per copy of the minor allele. In each analysis, for each log_2_ fold change *X*, all eQTLs with an absolute log_2_ fold change larger than *X* were considered, and the power was calculated as the percentage of those eQTLs for which the null hypothesis is rejected at FDR ≤0.05.
(TIF)

Table S1 **Table of all significant eQTLs.** This table contains the complete results for all significant non-diseased human heart eQTLs (FDR ≤0.05). It contains for each SNP-transcript pair the SNP ID, gene or transcript IDs (HGNC, Entrez Gene, RefSeq), genomic locations, minor and major allele, minor allele frequency, beta (effect size per copy of the minor allele), p-value and distance between SNP and gene. The table is sorted on HGNC official gene symbol.
(XLS)
