# Supplementary figures and images for: Genome-Wide Identification of Expression Quantitative Trait Loci (eQTLs) in Human Heart
Source: PLoS One. 2014 May 20;9(5):e97380. doi: 10.1371/journal.pone.0097380 (PMC4028258; doi:10.1371/journal.pone.0097380)

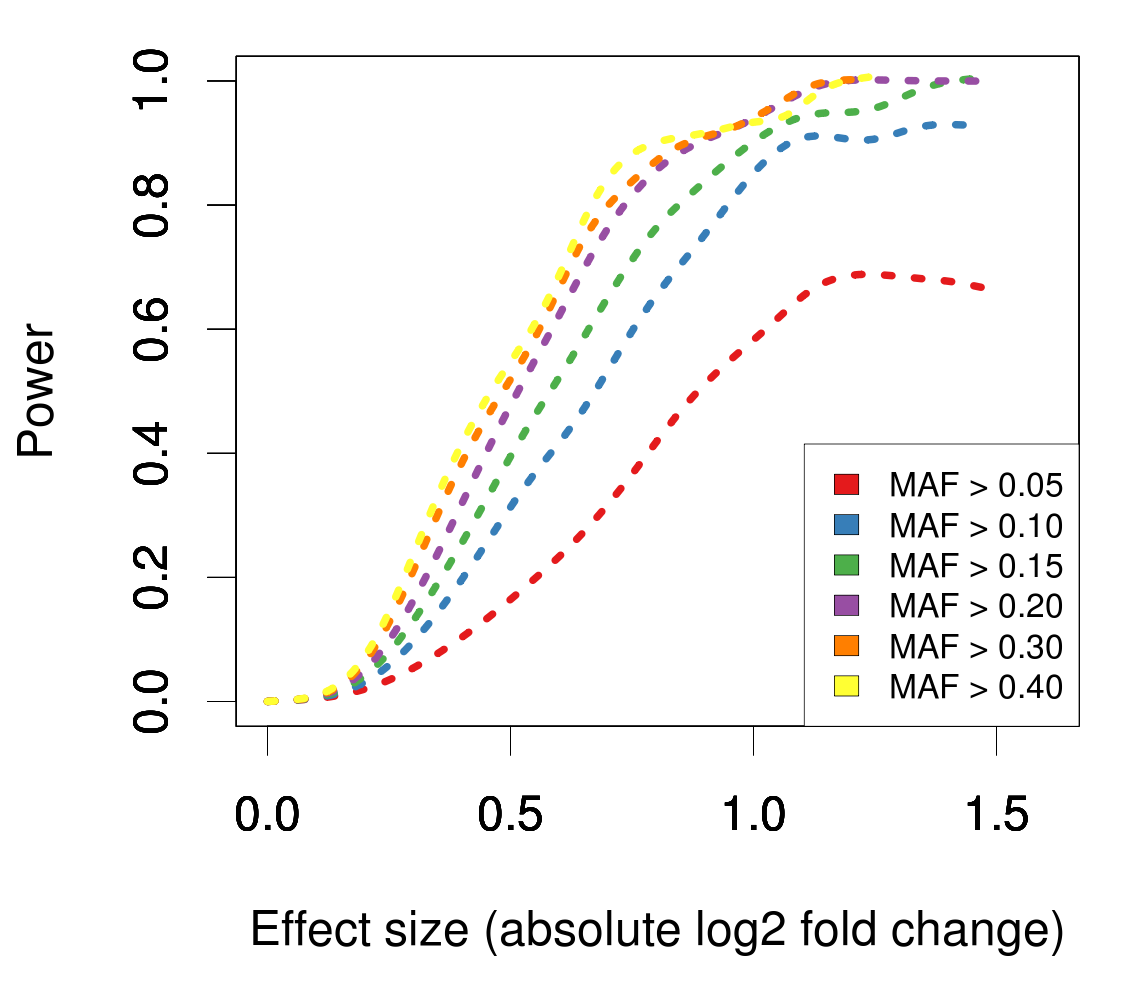

Supplement: Figure S2 — Results of eQTL power analyses in relation to MAF and gene expression fold change. eQTL power analyses were performed for different minimum minor allele frequencies (0.05, 0.10, 0.15, 0.20, 0.30 and 0.40). The gene expression fold change is defined as log2 difference in gene expression observed per copy of the minor allele. In each analysis, for each log2 fold change X, all eQTLs with an absolute log2 fold change larger than X were considered, and the power was calculated as the percentage of those eQTLs for which the null hypothesis is rejected at FDR ≤0.05. (TIF) [file pone.0097380.s002.tif]
